# Experience of anxiety among patients with severe COPD: A qualitative, in-depth interview study

**DOI:** 10.1017/S1478951513000369

**Published:** 2014-12

**Authors:** Susann Strang, Ann Ekberg-Jansson, Ingela Henoch

**Affiliations:** 1 Angered Local Hospital, Gothenburg, Sweden; 2 The Sahlgrenska Academy, Institute of Health and Care Sciences, University of Gothenburg, Gothenburg, Sweden; 3 The Sahlgrenska Academy, Institute of Medicine, University of Gothenburg, Gothenburg, Sweden

**Keywords:** COPD, Anxiety, Existential, Palliative care, Death, Coping

## Abstract

**Objectives:**

Anxiety often arises in conjunction with dyspnoea in patients with severe COPD. Considering the provoking symptomatology and the high mortality rate for COPD, it is reasonable to believe that these conditions trigger death-related and existential anxiety. Although anxiety causes considerable distress and reduces quality of life, people's experience of anxiety has been studied relatively little. The aim of this study was to explore severely ill COPD patients’ experience of anxiety and their strategies to alleviate anxiety.

**Methods:**

This qualitative, in-depth interview study explored perceptions of anxiety and the alleviation strategies that are adopted. Interviews were analyzed using a thematic content analysis approach, involving interpretive coding and identification of themes. People suffering from COPD (stage III or IV) were recruited from a pulmonary outpatient clinic in the west of Sweden. Purposive sampling was used, and thirty-one (31) patients were included.

**Results:**

Most of the patients had experienced anxiety associated with COPD. Analyses revealed three major themes, death anxiety, life anxiety, and counterweights to anxiety. Death anxiety included fear of suffocation, awareness of death, fear of dying and separation anxiety. Life anxiety included fear of living and fear of the future. Counterweights to anxiety concerned coping with suffocation, avoiding strategy, and a sense of joy that defied their vulnerable situation.

**Significance of results:**

The majority of patients experienced anxiety, which limited their lives. Although the patients experienced both life anxiety and death anxiety, they were able to cope with the situation and find a defiant joy to some extent.

## INTRODUCTION

COPD is nowadays a common cause of death throughout the world and an estimated 64 million people had COPD worldwide in 2004 (WHO, 2004). COPD is a progressive disease with only minor symptoms in the beginning. However, once the disease enters a more severe stage a substantial level of suffering associated with COPD occurs, such as shortness of breath, fatigue, depression, malnutrition, and limitations in daily living (Gardiner et al., 2009; Elkington et al., 2004; Gysels et al., 2007).

Anxiety often appears together with dyspnoea in patients with COPD. The burden of these symptoms is comparable or even worse in patients with severe COPD than in patients with cancer or heart failure (Pantilat et al., 2012; Gore et al., 2000; Bausewein et al., 2010).

Anxiety is a state of unease and worry about real or perceived future events and the source of the threat is more elusive. Fear is normally perceived as the emotional reaction to a specific, identifiable threat and promotes self-defense, associated with the “fight or flight” response. Although the words “fear” and “anxiety” are frequently used interchangeably (Lucchetti et al., 2012), there is a clear distinction between these terms in palliative care practice.

According to Condrau, people think paradoxically about death, experiencing both anxiety and fascination. Condrau states that death anxiety has many facets, including being afraid of suffering, thoughts about what happens before death or afterwards and a fear of separation or total isolation (Jacobsen, 2008). Yalom regards the fear of death as a powerful force for our inner self. Although the fear of death appears seldom in its most obvious form, it still forces human beings to develop various defenses to overcome it (Yalom, 1980).

Life anxiety is described by Høgh Olesen as the fear of the possibilities and choices in life and the fear of the unpredictable and unknown (Jacobsen, 2008). Cullberg (2003) argues that life anxiety is associated with the life changes that give rise to ideas about the meaning of life and death and demands new formulations of our existence.

Considering the severe symptomatology and that COPD is a disease with a high mortality rate, it is reasonable to believe that these conditions activate an urgent desire to live and that they trigger death-related and existential anxiety and unsettle our basic assumptions about life, i.e., the world as a secure place (Adelbratt & Strang, 2000; Ek & Ternestedt, 2008; Yalom, 1980; 2008; Johnson et al., 2011).

Because of a less predictable prognosis compared to disseminated cancer and the lack of recognition of the need for palliative care services in patients with COPD, they are not offered palliative care as a matter of routine. Death, dying, anxiety, or advance care planning are seldom discussed in the care of patients in the severe stages of COPD (Curtis et al., 2005; Reinke et al., 2011). Furthermore, there is a lack of recognition of existential needs in conjunction with COPD (Elofsson & Ohlen, 2004; Ek & Ternestedt, 2008), despite the fact that it is a life threatening disease associated with anxiety-provoking symptoms.

Although there are studies that show that patients with COPD suffer from anxiety, there has been limited examination of what this anxiety entails. The aim of this study, therefore, was to explore the nature, sources, and consequences of anxiety in patients with severe COPD and the strategies they adopt to alleviate anxiety.

## METHODS

### Patients

Individuals with COPD were recruited from a pulmonary outpatient clinic in an area in the west of Sweden with high smoking rates and low socioeconomic status. Purposive, maximum variation sampling was used with regard to participant gender, ethnicity, and age. Interviews were conducted until data saturation was reached, with a final sample of 31 patients (Patton, 1990). The inclusion criteria were: over 18 years, suffering from COPD according to GOLD stage III or IV, and with no obvious psychiatric diagnosis (COPD, 2011). The characteristics of the participants are summarized in Table 1.

**Table 1.** Demographic data. Descriptive information of interview participants

Nr	Gender	Age	Gold stage	FEV1% predicted	Smokers/ ex.smokers	Social background	Martial status
1	Female	81	3	48	Ex.smoker	Blue collar/pensioner	Widow
2	Female	70	4	unknown	Ex.smoker	Blue collar/ pensioner	Married
3	Male	67	4	unknown	Ex.smoker	Blue collar/ pensioner	Married
4	Male	75	3	54	Ex.smoker	Blue collar/ pensioner	Married
5	Male	76	4	19	Ex.smoker	White collar/ pensioner	Married
6	Female	62	4	35	Ex.smoker	Blue collar	Single
7	Female	71	4	22	Ex.smoker	Blue collar	Widow
8	Female	70	4	27	Ex.smoker	Blue collar/pensioner	Widow
9	Female	76	4	30	Ex.smoker	Blue collar/ pensioner	Widow
10	Male	56	3	43	Ex.smoker	Blue collar	Married
11	Female	81	4	unknown	Ex.smoker	Blue collar/ pensioner	Single
12	Male	85	4	unknown	Ex.smoker	White collar	Single
13	Male	75	3	19	Ex.smoker	Blue collar/ pensioner	Single
14	Female	48	4	26	Ex.smoker	Blue collar	Divorced
15	Female	62	3	34	Ex.smoker	Blue collar	Divorced
16	Female	59	3	47	Ex.smoker	Blue collar/Sickness benefit	Single
17	Female	64	4	22	Ex.smoker	Blue collar	Married
18	Female	68	4	24	Ex.smoker	Blue collar/ pensioner	Divorced
19	Male	73	4	25	Ex.smoker	Blue collar/ pensioner	Widowed
20	Female	63	4	27	Ex.smoker	Blue collar/ Sickness benefit	Widow
21	Male	68	3	35	Ex.smoker	Blue collar/ pensioner	Single
22	Male	85	3	44	Ex.smoker	Blue collar/ pensioner	Single
23	Female	66	3	39	Ex.smoker	Blue collar/ pensioner	Single
24	Male	64	3	32	Ex.smoker	Blue collar	Single
25	Female	70	3	41	Ex.smoker	Blue collar/ pensioner	Single
26	Male	81	3	54	Ex.smoker	Blue collar/ pensioner	Married
27	Female	60	3	49	Ex.smoker	Blue collar	Married
28	Male	67	4	25	Ex.smoker	Blue collar/ pensioner	Married
29	Male	78	4	27	Ex.smoker	White collar/ pensioner	Single
30	Male	66	3	55	Ex.smoker	Blue collar/ pensioner	Married
31	Male	60	3	50	No. smoker	Blue collar	Married

### Interview

Face-to-face interviews were conducted between June 2011 and March 2012. They lasted 17–76 minutes and took place either at the patients’ homes or at the clinic. The short duration of some interviews was due to the patients’ tiredness. The main interview question in this study was: “Can you describe what living with COPD means to you?” Additional questions were: “What are your experiences of anxiety?” “How do you view your future?” “What gives your life joy?” The interviews were recorded and transcribed verbatim and notes were taken to support the verbal information.

### Data Analysis

In this qualitative, descriptive, and interpretative study, manifest and latent content analysis was used to obtain patients’ perspectives on living with COPD. The manifest level concerns the obvious parts of the text and the latent level comprises an interpretation in which deeper aspects of meaning are sought in the text (Graneheim & Lundman, 2004; Downe-Wamboldt, 1992). The analysis was performed in several steps with no predetermined categories (Karlsson et al., 2012; Graneheim & Lundman, 2004, Krippendorff, 2004). In the first step, the interviews were read through and listened to several times to gain a sense of the whole and to become familiar with the individual interviews. The text was then divided into units of meaning, which were condensed and labeled with codes. These were sorted and abstracted into three themes and nine sub-themes (Table 2). Coding and development of categories were carried out mainly by the first author whilst the co-authors focused on validation of the results.

**Table 2.** Example of a unit of meaning and Code, sub-theme and theme

Meaning unit	Code	Sub-theme	Theme
To be honest, I wonder if I live until next summer. I think I'm getting worse and worse and everything gets more and more difficult. This body cannot keep on panting and breathing in this way, like I do for hours sometimes. No 28	Will I live another year? My body can't go on anymore	Awareness of dying	Death anxiety
I can feel numb all over. The seizures make me so tired and I think that now I must help from someone. I'm just sitting at home, fighting until the ambulance arrives and they can help me. And I actually think that I'll die because I can't get any air. *I: How does it feel?* A lot of anxiety. Yes, there's a lot of anxiety. Well, I don't know how to put it; I'm afraid of dying. It's no fun fighting on your deathbed and not being able to breathe. That would be terrible. Many times I think; just as long as I don't die of COPD. No 25	Becomes paralyzed by COPD attacks Believes that you may die as a result of COPD attacks Experiences anxiety at attack Fear of dying of COPD	Fear of suffocation	Death anxiety

### Ethical Considerations

Ethical approval of the study was obtained from the Regional Ethical Review Board in Gothenburg, (no. 209-11)**.** The participants were provided with verbal and written information and they gave informed consent.

## RESULTS

Three major themes emerged in the analyses; death anxiety, life anxiety, and counterweights to anxiety (Table 3).

**Table 3.** Overview of the themes and sub-themes

Themes	Sub-themes
**Death anxiety**	Fear of suffocation
	Awareness of death
	Fear of dying
	Separation anxiety
**Life anxiety**	Fear of living
	Fear of the future
**Counterweight to anxiety**	Coping with suffocation
	Avoiding strategy
	The defiant joy

### Death Anxiety

#### Fear of Suffocation

Most patients had experienced anxiety associated with COPD. The most obvious anxiety and panic were associated with acute respiratory distress. Patients felt as if they were being suffocated and strangled. The feeling of not getting enough air led to a fear of death. This fear limited the patients’ daily lives and created insecurity since they never knew when the next anxiety attack would occur.

It felt just like I was about to die. It's that damned fear when you can't get enough air. It's so hard to imagine. Not being able to get enough air. It feels like I'm suffocating. No. 7.

“I'm afraid to go out. The last time I went out I thought, oh my God, is this it? I'm going to die (laughs) … Couldn't get any air. You don't get … cramp but you become completely … How can I describe it … Anxious // When it passes I take Ventolin and wait. Yes then everything's fine again … for a while. No. 8.

#### Awareness of Death

The patients also experienced death anxiety, which was not related in the first instance to shortness of breath but to the fact that COPD had transformed death into a real threat and the realization that they would be shortly forced to leave life itself. When the patients were asked what they expected their lives to be like in one year's time, most patients doubted that they would still be alive as they realized that the disease is incurable.

Interviewer: How do you view your life one year from now?

Interviewee: Oh, by then I will not be alive! No. 16.

The insight that the disease would actually lead to death was difficult to deal with and gave rise to anxiety and many troubling thoughts about why it should happen to them.

“I'm afraid of the situation. I'm worried something bad will happen. It's no fun when you've fallen flat on your face on the square. This causes great anxiety and then the anxiety is replaced by sadness. Since I became ill I think about what will happen. What will I do and what will it be like? No. 22.

#### Fear of Dying

A number of the informants experienced anxiety and fear about the actual process of dying, such as what will precede death, whether they will be choked to death and what happens after death. Many patients had experienced close encounters with death during periods of acute dyspnoea and there was an obvious fear that the dying process would be a long struggle. Two patients expressed fear of being pronounced dead prematurely and being buried alive. One patient expressed anxiety about the funeral but not about death itself.

“These problems that I have now may lead to a situation where I slowly choke to death, struggling for air, with intense anxiety and all that stuff. It's obviously something you would rather not experience. So there's a vast difference between **dying** and **how you die**.” No. 13.

#### Separation Anxiety

A few patients experienced separation anxiety, manifested as concern about having to leave their children and spouses. This was extremely painful, especially for those with younger children.

Yes, I am afraid that I'll die. It goes around in my head: What will happen to my son I wonder. He's 12 years old. He says: 'Mum, I want you to live all the time', but I know that I'm so close to death. No. 19

### Life Anxiety

#### Fear of Living

A number of the informants described their anxiety as life anxiety, i.e., that the prospect of *living* and struggling with breathlessness and other COPD symptoms created more anxiety than the thoughts about *dying* from it. COPD restricted the patient's lives; they had lost much of their former self and their role in life, and they lived with both physical and mental limitations that led to a distressing existence.

That's why I'm so anxious the day I go to see the doctor. I don't know what will happen. It's spinning around in my head a week before the appointment. And the day I see the doctor … that night — I can't sleep at all.” No. 10.

The physical losses, such as shortness of breath, fatigue, and lack of energy, made it extremely exhausting and sometimes impossible to perform everyday tasks, such as housekeeping, walking, car maintenance, gardening, or pursuing hobbies. They were afraid to live their lives as they used to and they refrained from doing ordinary things.

“It's lonely living with this disease. Ordinary people have difficulty understanding that you become so physically tired and are unable to take part in ordinary activities. They say, get out and move around. But I can't! Just walking to the kitchen makes me breathless. It's like sitting in prison. No. 31.

This leads to a feeling of being imprisoned in one's home and in one's own body. This was exemplified by one participant, who had no energy due to COPD. She felt that other people commented on how slow she was and someone said to her when she was out on her bike: “Is the snail out cycling?” She felt that this slowness was not only a physical problem; it also affected her whole self-esteem and even her entire life. These types of losses and limitations led to considerable anxiety in everyday living and triggered anxious thoughts: “Will I manage to live?” “How will I cope with all this?”

#### Fear of the Future

The patients felt that somehow they could manage the disease at the moment but with further deterioration it would be impossible for them to continue living. They expressed anxiety about ending up as a vegetable and fading away slowly or suffocating. These dark thoughts and fear about the future gave rise to anxiety in the present.

Interviewer: What are your thoughts about the future? Interviewee: Yes, you think it should be better. I don't want it to be like this. We're doing fine at home and everything. But the thoughts appear sometimes. Will I end up being a damn vegetable? That's not much of a life. Sometimes I have terrible anxiety. Oh my God! No. 4.

There were also contradictory statements about a strong will to live in contrast to a willingness to die and avoid life-sustaining measures.

Although some days were relatively good, living with uncertainty made it impossible to plan for the next day because they did not know what their health would be like from one day to the next. Simple things, such as going to the shop or meeting a friend, could be prevented due to the uncertainty of when the next attack would occur.

### Counterweight to Anxiety

#### Coping with Suffocation

Experiencing anxiety is difficult to endure and the patients sought ways to alleviate it. When suffocation as a source of anxiety took hold of the patients, they trusted in the health care service and they were eager to use medication, inhalers, and oxygen, although these were only useful to a limited extent. Many received information about prophylactic care through their medical contacts — their doctor and their physiotherapist — as well as courses for patients with COPD. Almost no one received palliative care. The patients also placed hope in new treatments, better medication, surgery, stem cell treatment, or lung transplants.

“We breathed together and did as we had been taught by the health care professionals and things like that. Then they prescribed these tablets for me to help me relax, which helped a lot.” No. 11.

The patients also trusted in their own ability and used their own strategies to deal with suffocation, such as sitting down, breathing exercises, waiting for the anxiety to pass, concentrating only on themselves, opening the window and avoiding stress. Some patients were driven by a strong sense of curiosity about life and they had trust in their own ability to improve their health. A few planned to start exercising and some patients put their hope in therapies such as eating badger fat and drinking goat's milk. Three patients did not expect any deterioration. They had had a good life and simply stated: “We all have to die sometime.”

#### Avoidance Strategy

When anxiety was of an existential nature and thoughts about death came over them, the patients tried somehow to encapsulate it, displace it, and avoid talking about the future, sleep away the time, or laugh off this difficult subject. Instead, they emphasized the importance of living for the moment, taking one day at a time, and taking advantage of what is here and now. Patients did not talk to their fellow patients or health care professionals about matters related to the future or death.

Interviewee: “No, I don't want to talk about death!”

Interviewer: “Well, then we'll leave the subject.”

Interviewee: “But of course, you have to die sometime. But now I mustn't think about death because my daughter has a little son, John.” (Laughs) No. 27.

## THE DEFIANT JOY — A SOURCE OF MEANING

Although life with COPD was perceived as heavy and distressing, there were also factors that gave life joy and meaning, especially good relationships with family, children, grandchildren, and friends. The telephone was an essential means of keeping in touch when they did not have the energy to meet people. Many mentioned their dog as being the most meaningful creature in their life. The dog not only provided company but also gave them a reason to go for a walk. One patient felt that having a dog made it even easier to stop smoking.

Hobbies and pastimes were other important elements that gave life meaning. It could be watching television, managing to work or performing simple household chores, such as dusting, washing, or shopping. It could involve pursuing their interests or participating in community activities. Many of the patients had actually re-evaluated their lives and now appreciated small, simple things, such as being able to get out of bed.

“I cannot do as much anymore; I can no longer go for a walk. But I sit by the window and look out at times. And I watch a lot of television, which I enjoy.” No. 5.

All the patients wanted to continue living and no one had any active thoughts about suicide or euthanasia.

“I often think about death because I have difficult times. It's hard to breathe, it's not easy living with COPD. I've no energy. I'm not afraid of dying. I've lived my life. But I don't want to die yet!” (Laughs) No. 30.

It was remarkable that when the patients were asked about what gave their lives meaning today, many talked about what had given their life meaning in the past, prior to becoming ill. In the light of the things they had lost because of the disease, many felt that their previous sources of joy no longer existed. Despite this, many still hoped to be able to get out into the fresh air, to be able to do errands or that tomorrow might be better.

## DISCUSSION

The results showed that experiencing anxiety was the rule rather than the exception, although the identification of anxiety is something of challenge since anxiety seems to be mixed with other types of psychological distress, such as depression, worry and negative affects (Kolva et al., 2011). This was obvious when patients felt as if they were suffocating, which is an extremely unpleasant experience, and that suffocation led to anxiety. Notably, even when the choking sensation had passed, patients still felt anxious. Living with severe COPD created a sense of helplessness and anxiety about how to be able to cope with life and all its challenges. The participants felt trapped; imprisoned inside their body. This situation can be described as life anxiety, since the very foundation of life was being shaken and living with COPD shrank their life to such an extent that it overwhelmed their existence entirely (Jacobsen, 2008; Ng et al., 2007).

Medication, education, and good health care are generally available for people for whom suffocation is a source of anxiety (Coventry, 2009). Even though anxiety due to breathlessness was extremely frightening, patients had found ways to handle it. According to Folkman's coping theory, patients used problem-focused coping with tangible advice on resistant breathing: do not rush, take the medication, and put into practice advice from the COPD course in order to relieve the anxiety and breathlessness (Coventry, 2009; Folkman, 2010). When anxiety related to life fading away, the patients used emotion-focused coping. Unmasked anxiety is difficult to bear (Adelbratt & Strang, 2000; Yalom, 1980) and a common way was to avoid talking about difficulties or to think positively.

There is reason to believe that the patient uses the same models to cope with an existential crisis as a physical crisis. When health care staff “treats” anxiety, the focus is mostly on anxiety caused by suffocation. The reason for patients to seek care is usually physical deterioration, although they are also likely to do so in cases where the real reason is “life anxiety,” i.e., a fear of the unpredictable and the unknown (Jacobsen, 2008). This might explain the example of the clinical observation that patients with advanced COPD often use high doses of bronchodilating agents all day long and not just in acute situations. Short-acting beta-agonists have minimal bronchodilating effect, but in high dosages they also cause a rapid heart rate and palpitations. These sensations might even increase the physical experience of anxiety. If treatment and coping strategies were adapted more to life anxiety, this might reduce the patients’ suffering and the need for overdosing short-acting beta-agonists (Coventry, 2009).

Despite the hardships, there was still joy that defied everything else and they wanted “another summer.” It was clear that COPD patients found a new meaning and derived pleasure from small, everyday things, such as being able to dusting or to go shopping — things they previously took for granted. This can be expressed as meaning-focused coping (Folkman. 2010), or in part as a response shift (Sprangers & Schwartz, 1999), but is probably related more to a human being's inherent desire for life (May, 1983). It also emerged clearly that all patients wanted to continue to live despite severe symptoms and none of them wanted to end their lives. This is in line with an earlier study, which shows that patients suffering from severe diseases do not advocate euthanasia for themselves (Karlsson et al., 2012).

Almost all patients expected a sharp deterioration in the near future and they were not sure if they would survive another year. Yet these groups of patients are rarely included in palliative care (Hardin et al., 2008). Patients with COPD have a longer “palliative phase” with severe symptoms, including anxiety (Bausewein et al., 2010). Nevertheless, studies showed that the symptoms in COPD patients, such as anxiety and depression, are rarely addressed and the possibility of having medication prescribed is less than in other groups with life-threatening diseases (Leah, 2010; Cafarella et al., 2012; White et al., 2011).

Good communication and genuine presence is one vital way to make the disease comprehensible (Booker, 2005; Kissane et al., 2012). For instance, a patient might have a generalized death anxiety, although in a dialogue the anxiety is found to mainly concern the prospects of being choked to death. Then anxiety can be transformed into manageable fear, which is less frightening and easier to handle than anxiety (Yalom, 1980). The fear can then be reduced, for example, by providing information about the normal dying process and telling them that with good palliative care they would not need to choke to death.

## LIMITATIONS OF THE STUDY

In all qualitative studies, the samples are not statistically representative and the results cannot be generalized to a larger population, but they could be of help in understanding similar situations. To minimize the researchers’ subjectivity when analyzing data, two nurses and one respiratory physician participated in order to increase reflection.

A limitation is that the interviews were conducted at just one point in time. Due to the severity of the patientś illness, it was not possible to conduct repeated interviews. A few patients who suffered from severe fatigue were too tired to undergo a full interview and consequently the number of informants was extended to 31.

## IMPLICATIONS FOR SUPPORTIVE AND PALLIATIVE CARE

The majority of patients experienced anxiety, which limited their lives. Anxiety and impending death are seldom discussed in COPD care, which leaves patients alone to cope with their burden. If we increase our knowledge in this area, we will have the opportunity to satisfy palliative care needs in the growing population of patients suffering from COPD and thus have the opportunity to decrease both life anxiety and death anxiety. Future investigations into how to provide optimal support for COPD patients with anxiety are warranted.
